# Current News

**Published:** 1903-07

**Authors:** 


### Current News.

### REPORT OF THE COMMITTEE ON CONGRESSES: FOURTH INTERNATIONAL DENTAL CONGRESS, ST. LOUIS, 1904.

In the matter of the organization of the Fourth International Dental Congress, designated by the International Dental Federation to be held in Saint Louis, August, 1904, the Committee on Congresses for the Exposition find the following facts and reach the subjoined conclusion:

At the meeting of the National Dental Association of the United States at Niagara Falls, in 1902, a communication was forwarded to the International Dental Federation at Stockholm to designate the St. Louis Exposition as the place and August, 1904, as the time for holding the Fourth International Dental Congress. This communication was conveyed by a Committee of Three to Stockholm. On the day following the departure of the committee the National Dental Association appointed a Committee of Fifteen to organize and conduct the Congress, subject to the acceptance of the invitation by the International Dental Federation.

The International Dental Federation accepted on the 18th of August, 1902, at Stockholm, the invitation of the National Dental Association, of the governor of the State of Missouri, the mayor of the city of St. Louis, the Universal Exposition of St. Louis, and various dental societies, and decided that the Fourth International Dental Congress should be held in the month of August, 1904, in the city of St. Louis. They further appointed a Committee of Nine—all Americans—to assist in the organization of the Congress.

A question then arose as to which of the committees thus appointed—the Committee of Fifteen or the Committee of Nine—had the power to organize the Congress. Relative to this question also came up the point as to the official connection between the National Dental Association and the International Dental Federation. On this point there is much divergence of opinion. The facts seem to be established, from the minutes of the National Dental Association, that at the Omaha meeting on August 30, 1898, a Committee of Nineteen was appointed to represent the National Dental Association at the Third International Dental Congress in Paris, 1900; that this official committee took part in the Third International Dental Congress, at which the International Dental Federation was formed; that the duties of this Federation, as set forth in the minutes of the Paris meeting, were the appointment of an International Commission on Instruction and to prepare for the next International Dental Congress; that the National Dental Association of America, at their meeting in Milwaukee in August, 1901, received the report of the Chairman of the Committee of Nineteen for the International Congress at Paris, 1900, which report contained an account of the formation of the International Dental Federation and the part said committee played therein. They accepted the report and ordered it printed.

Whether or not the acceptance of this report, which seems to have been made in the language ordinarily used in the submitting of similar reports, legally binds the Association to a recognition of the Federation has been the point under discussion. The language used on page 239 of the Transactions of the National Dental Association for 1901 is, "On motion, it was accepted and ordered printed." The report was included in the minutes, which were read and approved at the next day's meeting. On page 238 a similar phraseology is used in reference to another report. On pages 222 and 228 the same occurs. On the latter page, also, the Annual Report of the Treasurer was "received and, on motion, accepted." From this it would appear that the National Dental Association is a member of the Federation. This view would also seem to be recognized by the National Association itself in forwarding to Stockholm the invitation to hold the Fourth International Congress in St.

Louis, the invitation reading: "The National Dental Association of the United States of America hereby invites the Committee of the International Dental Federation *having in charge the promotion of the Fourth International Dental Congress,"* etc.; and further in the appointment of a Committee of Fifteen, that should be provisional only until said invitation was accepted.

But this does not seem to us at this juncture to be the crucial point. Had the International Dental Federation instructed the Committee of Nine definitely as to their duties, or had they, in response to the joint communication of the two committees in November, ruled positively on the point; or had they, in response to our communication to them at Madrid, laid down the procedure of the Federation, the decision would be easily arrived at.

For want of such action we are obliged to consider the matter purely from the stand-point of precedent and exposition expediency.

To resume the findings of facts: The Committee of Nine was appointed by the Federation at Stockholm presumably without knowledge of the appointment of the Committee of Fifteen. Unfortunately the powers of the Committee of Nine are not set forth definitely and precisely, either in the official acceptance of the invitation or in the letter of appointment to the individual members.

A praiseworthy attempt was made by the two committees at Cincinnati to combine their work, but was refused on the part of the Committee of Nine on the ground that such amalgamation was not within their instructions. The matter was then referred, in a joint note from the two committees, to the Chairman of the Executive Committee of the Federation for decision, but the answer was returned that he did not consider himself authorized to select between the two committees, and consequently was obliged to refer the answers to the questions to the next session of the International Dental Association, to be held in Madrid in April, 1903.

On February 6 the president of the Exposition appointed as an organizing committee for the Fourth International Dental Congress on the part of the Exposition, the Committee of Fifteen of the National Dental Association, following the precedent of this and other expositions of making national associations responsible for the success of the congresses. The powers of the committee were, however, left in abeyance pending decision of the questions hereinbefore set forth. As a result of the various meetings and of much correspondence, the Exposition sent to the Madrid meeting, by a special delegate, a communication,—copy of which is hereto attached,—particular attention being called to the phraseology of the instructions to the Committee of Nine on the part of the Federation, and an interpretation asked therefor. The reply of the Federation to this communication, which has just been received, does not meet these points, but rather suggests an amalgamation of the two committees, the Committee of Nine having the power to raise its representation to the numerical equality of the Committee of Fifteen.

We are convinced that it is impossible, in view of the present status of the discussion, to make, at the present time, an effective consolidation of the two committees. We are sure that the International Dental Federation would agree with us were they in closer touch with the situation. So large a committee is also impractical from an administrative stand-point. There seems to be nothing left in order to create a successful congress except for the Exposition Company to define the duties of the Committee of Organization of the Exposition, and suggest the line of co-operation of the Committee of the International Dental Federation.

The fact that the International Dental Federation does not specifically set forth the duties of the committee appointed by it, and hesitates after various appeals to interpret their instructions to the committee, would seem to imply that there was no settled line of policy on such matters, and that the Federation does not desire, without further authority from an international congress, to formulate one.

In the absence of such definite statement as to procedure, it would seem to devolve upon the Universal Exposition of 1904 to follow the precedents heretofore established and organize the Congress, through its duly appointed committee, depending upon the International Dental Federation, through its committee, to interest alike the dentists of all countries and to secure international cooperation. As to the precedent to be followed, we call attention to the action of the Second International Dental Congress at Chicago, in 1893, whereby the organization was continued to the Paris Exposition of 1900. The authorities of the latter Exposition assumed the right to appoint a National Committee of Organization, which provided the ways and means, issued the invitations, appointed the committees, and nominated the officials of the Third International Congress.

We conclude that, in view of the nearness of the Exposition, and the necessity of immediate organization, and in view of the impossibility of amalgamating the two committees, it is imperative to give the Committee of Organization of the Exposition the power to proceed energetically without further delay. We would respectfully suggest that this is in no wise to be considered a precedent for future expositions, or an attempt to define the powers of the International Dental Federation or the National Dental Association, but an *action demanded by the emergency of the situation;* and we further recommend that at the time of the Congress in 1904 a definite form of procedure for holding future congresses be fixed.

We therefore instruct the Organizing Committee of the Exposition to proceed in the work of organizing the Congress, to obtain the ways and means therefor, to issue invitations, to appoint the necessary committees for preparing the programme, for the entertainment of guests, for nominations, etc., and on such committees to give equal representation in membership to the Committee of Nine, and in all matters to take due care of the rights of every foreign nation and of the obligations due to the International Federation as the authorizing body of the Congress.

(Signed) Howard J. Rogers,
*Director of Congresses.*

Approved:
Frederick W. Lehmann,
*Chairman Committee on Congresses.*

---

### TENNESSEE DENTAL ASSOCIATION.

The next meeting of the Tennessee Dental Association will be held at Lookout Inn, Chattanooga, Tenn., July 23, 24, and 25, 1903. The outlook is bright for the best meeting in the history of the Association. Contributions have been promised from many eminent in the profession from Tennessee and other States.

A one-and-one-third-fare rate on the certificate plan has been secured. Those wishing to attend the meeting of the National Dental Association at Asheville, N. C., can deposit return tickets with agent at Chattanooga and take up same on return trip. All members are urged to be present, and a cordial invitation is extended to all ethical dentists to be present and take part in the proceedings.

The Tennessee State Board of Dental Examiners will hold its meeting at the same time and place.

A. Sidney Page,
*Secretary.*

---

### ILLINOIS STATE DENTAL SOCIETY.

At the thirty-ninth annual meeting of the Illinois State Dental Society, held in Bloomington, May 12 to 14, 1903, the following officers were elected:

President, F. H. McIntosh, Bloomington; Vice-President, C. C. Corbett, Edwardsville; Secretary, Hart J. Goslee, Chicago; Treasurer, C. N. Johnson, Chicago; Supervisor of Clinics, C. E. Bentley, Chicago; Librarian, J. T. Cummins, Metropolis City.

*Executive Committee.*—F. B. Noyes, Chicago.

*Committee on Science and Literature.*—G. V. Black, Chicago.

*Committee on Art and Invention.*—J. H. Prothero, Chicago.

*Members of Executive Council* (three years).—A. H. Peck, Chicago; G. W. Gittmar, Chicago; W. A. Johnston, Peoria.

The fortieth annual meeting will be held in Peoria the second Tuesday in May, 1904.

Hart J. Goslee,
*Secretary.*

---

### CONNECTICUT STATE DENTAL COMMISSIONERS.

Governor A. Chamberlain, of Connecticut, appointed, on March 19, 1903, for Dental Commissioners to serve for two years from July 1, 1903, the old Commissioners, as follows: Dr. Edward W. Pratt, President, East Hartford; Dr. J. Tenney Barker, Recorder, Wallingford; Dr. William H. Loomis, Rockville; Dr. William E. Hyde, Danielson; Dr. Horace Bascom, New Haven.

On May 1 Dr. Wm. E. Hyde sent in his resignation, to take effect immediately, and Dr. Theo. S. Rust, of Meriden, was appointed for the unexpired term and for two years from July 1, 1903.

Dr. Horace Bascom also resigned, his resignation to take effect on July 1.

